# Plasma metabolite profile of legume consumption and future risk of type 2 diabetes and cardiovascular disease

**DOI:** 10.1186/s12933-023-02111-z

**Published:** 2024-01-20

**Authors:** Hernando J. Margara-Escudero, Indira Paz-Graniel, Jesús García-Gavilán, Miguel Ruiz-Canela, Qi Sun, Clary B. Clish, Estefania Toledo, Dolores Corella, Ramón Estruch, Emilio Ros, Olga Castañer, Fernando Arós, Miquel Fiol, Marta Guasch-Ferré, José Lapetra, Cristina Razquin, Courtney Dennis, Amy Deik, Jun Li, Enrique Gómez-Gracia, Nancy Babio, Miguel A. Martínez-González, Frank B. Hu, Jordi Salas-Salvadó

**Affiliations:** 1 Universitat Rovira i Virgili, Departament de Bioquímica i Biotecnologia, Alimentació, Nutrició, Desenvolupament i Salut Mental ANUT-DSM, Reus, Spain; 2 Alimentació, Nutrició, Desenvolupament i Salut Mental, Institut d’Investigació Sanitària Pere Virgili (IISPV), Reus, Spain; 3 Centro de Investigación Biomédica en Red Fisiopatología de La Obesidad y La Nutrición (CIBEROBN), Instituto de Salud Carlos III, Madrid, Spain; 4 Department of Preventive Medicine and Public Health, University of Navarra, Instituto de Investigación Sanitario de Navarra (IdiSNA), Pamplona, Spain; 5 Department of Nutrition, Harvard T.H. Chan School of Public Health, Boston, MA USA; 6 Department of Epidemiology, Harvard T.H. Chan School of Public Health, Boston, MA USA; 7 Channing Division of Network Medicine, Brigham and Women’s Hospital and Harvard Medical School, Boston, MA USA; 8 The Broad Institute of Harvard and MIT, Boston, MA 02142 USA; 9 Navarra Institute for Health Research, IdiSNA, Pamplona, Navarre Spain; 10 Department of Preventive Medicine, University of Valencia, Valencia, Spain; 11 Department of Internal Medicine, Institut d’Investigacions Biomèdiques August Pi Sunyer (IDIBAPS), Hospital Clinic, University of Barcelona, Barcelona, Spain; 12 Department of Endocrinology and Nutrition, Institut d’Investigacions Biomèdiques August Pi Sunyer (IDIBAPS), Lipid Clinic, Hospital Clínic, Barcelona, Spain; 13 Centro de Investigación Biomédica en Red (CIBERESP) de Epidemiología y Salud Pública, Instituto de Salud Carlos III, Madrid, Spain; 14 Cardiovascular Risk and Nutrition Research Group, Hospital del Mar Research Institute, Barcelona, Spain; 15 Department of Cardiology, University Hospital of Alava, Vitoria, Spain; 16 Illes Balears Health Research Institute (IdISBa), Hospital Son Espases, Palma, Spain; 17 Department of Public Health, Section of Epidemiology, University of Copenhagen, Copenhagen, Denmark; 18 Novo Nordisk Foundation Center for Basic Metabolic Research, University of Copenhagen, Copenhagen, Denmark; 19 Department of Family Medicine, Research Unit, Distrito Sanitario Atención Primaria Sevilla, Seville, Spain; 20 Preventive Medicine and Public Health Department, School of Medicine, University of Málaga, 29010 Malaga, Spain; 21 Biomedical Research Institute of Malaga-IBIMA Plataforma BIONAND, 29010 Malaga, Spain

**Keywords:** Metabolomics, Plasma, Cardiovascular disease, Type 2 diabetes, Legumes

## Abstract

**Background:**

Legume consumption has been linked to a reduced risk of type 2 diabetes (T2D) and cardiovascular disease (CVD), while the potential association between plasma metabolites associated with legume consumption and the risk of cardiometabolic diseases has never been explored. Therefore, we aimed to identify a metabolite signature of legume consumption, and subsequently investigate its potential association with the incidence of T2D and CVD.

**Methods:**

The current cross-sectional and longitudinal analysis was conducted in 1833 PREDIMED study participants (mean age 67 years, 57.6% women) with available baseline metabolomic data. A subset of these participants with 1-year follow-up metabolomics data (n = 1522) was used for internal validation. Plasma metabolites were assessed through liquid chromatography-tandem mass spectrometry. Cross-sectional associations between 382 different known metabolites and legume consumption were performed using elastic net regression. Associations between the identified metabolite profile and incident T2D and CVD were estimated using multivariable Cox regression models.

**Results:**

Specific metabolic signatures of legume consumption were identified, these included amino acids, cortisol, and various classes of lipid metabolites including diacylglycerols, triacylglycerols, plasmalogens, sphingomyelins and other metabolites. Among these identified metabolites, 22 were negatively and 18 were positively associated with legume consumption. After adjustment for recognized risk factors and legume consumption, the identified legume metabolite profile was inversely associated with T2D incidence (hazard ratio (HR) per 1 SD: 0.75, 95% CI 0.61–0.94; p = 0.017), but not with CVD incidence risk (1.01, 95% CI 0.86–1.19; p = 0.817) over the follow-up period.

**Conclusions:**

This study identified a set of 40 metabolites associated with legume consumption and with a reduced risk of T2D development in a Mediterranean population at high risk of cardiovascular disease.

*Trial registration*: ISRCTN35739639.

**Supplementary Information:**

The online version contains supplementary material available at 10.1186/s12933-023-02111-z.

## Background

Non-communicable diseases, such as cardiovascular disease (CVD) and type 2 diabetes (T2D) remain significant public health concerns, significantly reducing individuals' life quality, increasing healthcare costs, and resulting in premature death [1, 2]. Thus, there is an urgent need to establish preventive and treatment strategies for these diseases. In recent years, accumulating evidence from prospective studies and randomized controlled trials (RCT) suggests that the Mediterranean diet (MedDiet), which also integrates social behaviours, holds promise in preventing various cardio-metabolic diseases [3]. A relevant food component of the MedDiet is legumes (such as green beans, peas, broad beans, dry beans, chickpeas, dry peas, and lentils), which are important sources of vegetable-based proteins, soluble and insoluble dietary fibre, oligo and polysaccharides, unsaturated fatty acids, pyridoxine, folate, iron, zinc, calcium, flavonoids and lignans [4–6].

In the context of MedDiet, legumes are recommended to be regularly consumed (≥ 3 times/week) whether cooked, baked, raw, or as sprouts or salads, owing to their beneficial effects on health [6]. Indeed, incorporating legumes into a well-balanced diet in moderate amounts has been associated with a decreased risk of various health conditions, including hypertension, obesity, CVD and T2D, as well as with an improvement in markers of glycaemic control [7–10]. In addition, it has been shown that substituting red meat with legume consumption decreases peripheral inflammation, improves glycaemic control and reduces insulin levels in individuals with T2D [11]. Nevertheless, some studies showed inconsistencies regarding the association between legume consumption and diabetes, stroke, or coronary heart disease [7]. Methodological issues, such as the heterogeneity of the population studied or the misclassification arising from the use of food frequency questionnaires (FFQs) not specifically validated for legume consumption assessment [12], could partially explain discrepancies among studies.

Nowadays, the integration of omics sciences, such as metabolomics, in nutritional epidemiology shows immense potential in reducing measurement errors associated with self-reported dietary intakes. This integration will play a key role in enhancing the precision of dietary markers, mitigating inherent biases in the assessment of FFQs and improving the precision of estimation of the intricate interplay between diet and well-being [13–15]. Recent studies employing omics technologies have provided new insights into the potential biological mechanisms that explain the associations between various dietary components, such as walnuts, meat, fish, processed meat, dairy, wine, and coffee, with cardiometabolic health and T2D [16–20]. However, despite the extensive evidence demonstrating a wide range of health benefits of legume consumption, the biological mechanisms underlying these positive effects are not fully understood. Furthermore, to date, studies exploring the metabolic signature of legume consumption and its ability to predict the onset of diseases and progression are limited. Hence, we examined the associations between the metabolomic profile of legume consumption and the risk of CVD and T2D incidence. The findings may have the potential to enhance our comprehension of the mechanisms implicated in the observed links between legume consumption and the onset of CVD and T2D.

## Material and methods

### Study population

#### Discovery population

The present study was conducted within the framework of the PREDIMED study, a multi-centre, parallel-group RCT conducted in Spain from 2003 to 2010. The primary aim of PREDIMED (registered at http://www.controlled-trials.comb as ISRCTN35739639) was to investigate the impact of a traditional MedDiet on the primary prevention CVD in individuals with high cardiovascular risk. The details of the PREDIMED study protocol and its primary findings can be found elsewhere [21, 22]. All enrolled participants provided written informed consent, and the research protocol was approved by the Institutional Review Boards of all participating study centers.

The present analysis included participants from three studies conducted within the frame of the PREDIMED study. The first study focused on incident CVD as the primary event, the second study on incident T2D as a secondary end-point, and the third focused on a subset of PREDIMED participants who completed an oral glucose tolerance test (OGTT) at baseline. The PREDIMED-CVD study recorded 229 incident CVD cases free of CVD at baseline and 788 sub-cohort participants, with an overlap of 37 participants [23, 24]. Similarly, the PREDIMED-T2D study included 251 incident T2D cases and 694 sub-cohort participants without T2D at baseline, with an overlap of 53 participants. Finally, the OGTT study included 132 participants without T2D at baseline. The current analysis included participants with available baseline metabolomics data from these studies and had completed validated semi-quantitative 137-item FFQs, resulting in an initial sample size of 1882 individuals. Excluding participants with missing FFQ data (n = 11), implausible daily energy intake (< 500 or > 3500 kcal/d for women and < 800 or > 4000 kcal/d for men) (n = 34) at baseline, or those with missing values for ≥ 20% of metabolites (n = 4), the final sample for the present analysis included a total of 1833 participants at baseline (Additional file 1: Figure S1).

#### Validation population

The validation of the metabolite profile was performed using a subset of participants (n = 1522) from the initial discovery population (n = 1833). This subset was selected based on having repeated measurements of both dietary information and metabolomics data at baseline and 1-year follow-up. This sub-sample did not include individuals with incident cases occurring during the first year of follow-up.

### Dietary assessment

At baseline and after 1 year of follow-up, dietary intake was collected using a validated 137-item FFQ. Pearson correlation coefficient and intraclass correlation coefficient (ICC) were employed to assess the reproducibility of the semi-quantitative FFQ concerning food groups, energy, and nutrient intake [25]. Specifically, for legumes, the reproducibility and validity of the FFQ were found to be 0.47 (ICC 0.63) and 0.29 (ICC 0.40), respectively [25]. Information on legume consumption was assessed using four FFQ items (lentils, chickpeas, dry beans, and fresh peas). For the current analysis, total non-soy legume (as soya consumption was not recorded and was extremely infrequent in the PREDIMED population) was considered as the sum of the aforementioned legumes. The frequency of consumption was measured on a scale of nine categories, ranging from "never or almost never" to " > 6 servings/day”. The responses for each item were converted into daily frequencies and subsequently multiplied by the respective portion size, in order to obtain grams per day consumed during the follow-up. Two complementary Spanish food composition tables were used to estimate energy and nutrient intake [26, 27].

### Other measurements and covariates

At baseline, before randomization, and after 1-year of follow-up, participants completed a questionnaire on various aspects of their lifestyle, medical history, medication use, and a validated Spanish version of the Minnesota Leisure Time Physical Activity Questionnaire [28] to estimate leisure time physical activity. Additionally, trained personnel measured participants’ body weight, height, waist circumference, and blood pressure (in triplicate) according to the study protocol.

### Metabolite profiling

Plasma metabolomics profiling was performed at the Broad Institute of Harvard University and Massachusetts Institute of Technology using a combination of methods previously described [16]. In summary, 399 metabolites were selected for primary analyses after normality tests, quality filtering and standardization. Nonetheless, 14 metabolites were excluded as a consequence of a significant number of missing values (> 20%) or because were identified as drug metabolites (Acetaminophen, Atenolol, Cyclohexylamine, Metformin, Metronidazole, Valsartan, Verapamil, and Warfarin). Additionally, 3 metabolites were used as internal standards (1,2-didodecanoyl-sn-glycero-3-phosphocholine, valine-d8, and phenylalanine-d8) and excluded, resulting in a final dataset of 382 metabolites.

Metabolomics determinations were performed from plasma EDTA samples collected at baseline and at one year of follow-up. The samples were obtained after overnight fasting for at least 8 h, processed at each recruiting centre, and stored in −80 °C freezers. Pairs of samples from both cases and sub-cohort participants were randomly distributed before the analytical determinations.

Quantitative profiling of polar metabolites and lipids in plasma samples was carried out using high-throughput liquid chromatography-tandem mass spectrometry (LC–MS/MS). Complete methodology details of the LC–MS/MS have been published previously [29–31]. Amino acids (AAs) and other polar metabolites were profiled with a Nexera X2 U-HPLC system (Shimadzu) coupled to a Q-Exactive mass spectrometer (ThermoFisher Scientific) for this purpose. Samples (10 μL) were extracted using a mixture of 74.9% acetonitrile, 24.9% methanol, and 0.2% formic acid, which contained stable isotope-labeled internal standards (valine-d8 from Sigma-Aldrich and phenylalanine-d8 from Cambridge Isotope Laboratories). The extracted metabolites were separated on a 150 × 2-mm, 3-μm Atlantis HILIC column (Waters). Prior to injection, the samples underwent centrifugation (9000 × g; 10 min; 4 ºC) and the resulting supernatants were directly injected into the LC–MS/MS system. The column was subjected to an isocratic elution at a flow rate of 250 μL/min, using 5% mobile phase A (consisting of 10 mmol ammonium formate/L and 0.1% formic acid in water) for 0.5 min. This was followed by a linear gradient to 40% mobile phase B (comprising acetonitrile with 0.1% formic acid) for a duration of 10 min. The MS analyses were performed using electrospray ionization in the positive-ion mode and full-scan spectra were acquired in the m/z range of 70–800. Lipid profiling was conducted using a Nexera X2 U-HPLC (Shimadzu) coupled with an Exactive Plus orbitrap MS (Thermo Fisher Scientific). Lipids were extracted from plasma samples (10 μL) utilizing 190 μL of isopropanol, which included 1,2-didodecanoyl-sn-glycero-3-phosphocholine from Avanti Polar Lipids as an internal standard. Afterwards, 2 μL of the lipid extracts were injected onto a 100 × 2.1-mm, 1.7-μm ACQUITY BEH C8 column (Waters). The column was eluted isocratically with 80% mobile-phase A (containing 10 mM ammonium acetate/methanol/formic acid in a 95:5:0.1 vol:vol:vol ratio) for 1 min. This was followed by a linear gradient to 80% mobile-phase B (comprising methanol/formic acid in a 99.9:0.1 vol:vol ratio) over 2 min, followed by another linear gradient to 100% mobile-phase B over 7 min. Lastly, a 3-min hold at 100% mobile-phase B was performed. The MS analyses were conducted using electrospray ionization in the positive-ion mode with full-scan analysis in the m/z range of 200–1100. Raw data obtained from the MS analyses were processed using Trace Finder versions 3.1 and 3.3 (Thermo Fisher Scientific) and Progenesis QI from Nonlinear Dynamics. The identification of polar metabolites was confirmed using authentic reference standards, while lipids were identified based on their head group, total acyl carbon number, and total acyl double bond content. In order to assess data quality and ensure consistency across the analytical queue and sample batches, pairs of pooled plasma reference samples were analyzed at regular intervals of 20 study samples. One reference sample served as a passive quality control sample within each pair, enabling the evaluation of analytical reproducibility for measuring each metabolite. The other pooled sample was utilized for standardization through a "nearest neighbor" approach. To estimate standardized values, a ratio was established between the value of each metabolite in a given sample and the value of the nearest pooled plasma reference. This ratio was then multiplied by the median value measured across all the pooled references.

### Statistical analysis

Baseline characteristics of participants were presented according to tertiles of total legume consumption adjusted for energy intake using the residuals method [32]. For quantitative variables, mean and standard deviation (SD) were reported, while percentages (n) were provided for categorical variables. The selection and identification of metabolites associated with legume consumption were performed using plasma metabolomics data obtained from the PREDIMED study at baseline, serving as the training set or discovery population. The findings were self-validated using data from the PREDIMED study after 1 year.

Statistical quality control of metabolomic database were performed using the next method. Individual metabolites with less than 20% of missing values were imputed with random forest imputation method using the "missForest" function from the "missForest" R package, like previous publications [33, 34]. In addition, our research consortium has previously explored different alternatives to this approach, and it was found that these alternatives also produced consistent results [16]. Missing values in the dataset were attributed to determinations that fell below the limit of detection during the analysis process.

For the estimation of the metabolite profile, first, the data was centred and scaled using the SD as the scaling factor, a method known as autoscaling [34]. To address the issues of over-fitting and multicollinearity present in the data, gaussian linear and logistic regressions were employed with the elastic net penalty (R package “glmnet”) to build models of legume consumption. Elastic net regression was an appropriate approach due to the high dimensionality and collinear nature of the data. A tenfold cross-validation (CV) methodology was employed. The sample was divided into training and validation sets (90% for the training sample and 10% validation sample), repeated 10 times independently. Furthermore, within the training set, an additional tenfold CV was conducted to determine the optimal value of the tuning parameter [λ (lambda)] that minimizes the mean square error (MSE). The minMSE and minMSE + 1 standard error (SE) values were estimated using the argument s = “lambda.min” or s = “lambda.1se” in the cv.glmnet function (R package “glmnet”), respectively. In this case, alpha = 0.2 was the model with the best-predicting accuracy in the validation sets. Coefficients for each iteration of the tenfold CV were reported by employing the lambda.min in the elastic net linear regression. Moreover, for each pair of training-validation datasets, weighted models were constructed using the metabolite coefficients obtained from the elastic net regression applied to the training set.

For the self-validation of the metabolite profiles, Pearson correlation coefficients were determined to assess the relationship between self-reported legume consumption and the metabolite profile model in each pair of training-validation datasets. Correlation coefficients were reported based on 10 iterations of the tenfold CV elastic regression approach applied to the entire dataset. These analyses were based on consistency among cross-validation runs, and no p-values were derived. Additionally, correlations between legume-related metabolites and consumption of various food groups, including meat, fish, vegetables, fruit, cereals, dairy products, olive oil, nuts, alcohol and adherence to MedDiet were performed.

For the longitudinal analysis, Cox regressions with Barlow weights and robust variance estimator were employed to investigate the associations between the legume metabolite profile at baseline and after 1 year with incident T2D risk (245 incident cases at baseline and 161 events at 1 year) and CVD risk (222 incident cases at baseline and 151 events at 1 year) within the T2D and CVD nested case-cohort studies, respectively. The median time of follow-up of 3.7 years for T2D incidence and 3.8 years for CVD, was estimated as the interval between the baseline date and date of CVD or T2D event, death, or date of the last participant contact, whichever came first. For the internal validation analysis and for the T2D and CVD risk (1 year of follow-up), the participants who developed T2D or CVD during the first year of follow-up were excluded from this analysis. Multivariable model 1 incorporated adjustments for age, sex, and propensity scores, and were stratified by intervention group and recruitment center as potential confounding factors. Model 2 also included body mass index (BMI, kg/m2), smoking status (categorized as never, former, or current smoker), alcohol intake (g/d), squared alcohol intake (measured in grams per day), education level (classified as primary, secondary, or academic), physical activity (expressed in metabolic-equivalent minutes per day), family history of coronary heart disease (CHD) (indicated as yes or no), as well as consumption of meat, fish, vegetables, fruits, cereals, nuts, olive oil and dairy (g/day). Model 3 encompassed all adjustments from model 2 plus the consumption of total legumes (g/day). Propensity scores, as detailed in Estruch et al. [21], represent the likelihood of assigning participants to specific groups in the PREDIMED trial, such as the low-fat control group, the MedDiet with extra virgin olive oil, or the MedDiet with nuts. All statistical analyses were performed using R v. 4.1.2.

## Results

The present analysis included 1833 participants (57.6% women) with a mean age of 67 ± 6 years and with mean legume consumption of 20 ± 12 g/d. Baseline characteristics of the study population are summarized in Table 1. As compared with the lowest tertile of energy-adjusted legume consumption, those participants in the highest tertile had higher BMI and were more likely to have hypercholesterolemia. Regarding food group intake, individuals in the highest tertile reported higher dairy, vegetable, and fruit consumption, and a lower consumption of meat and cereals than those in the lower tertile. The general characteristics of participants at 1-year follow-up according to energy-adjusted tertiles of total legume consumption are shown in Additional file 1: Table S1.

**Table 1:** Participant’s general characteristics according to tertiles of energy-adjusted total legume consumption at baseline

Characteristics	T1 (*n* = 611)	T2 (*n* = 611)	T3 (*n* = 611)	Overall (*n* = 1833)
Total legume consumption (g/day)	11 ± 4	19 ± 2	32 ± 15	20 ± 12
Age (years)	67 ± 6	67 ± 6	68 ± 6	67 ± 6
Sex, women,* n* (%)	340 (55.6)	370 (60.6)	345 (56.5)	1055 (57.6)
Education level, *n* (%)				
Primary	431 (70.5)	473 (77.4)	457 (74.8)	1361 (74.2)
Secondary	114 (18.7)	86 (14.1)	102 (16.7)	302 (16.5)
Academic	56 (9.2)	40 (6.5)	37 (6.1)	133 (7.3)
Body mass index (kg/m^2^)	29.8 ± 3.6	29.7 ± 3.5	30.3 ± 3.6	29.9 ± 3.6
Waist circumference (cm)	100 ± 10	100 ± 10	101 ± 10	100 ± 10
Leisure time physical activity level (MET min/day)	253 ± 249	234 ± 217	246 ± 242	244 ± 237
Type 2 diabetes, n (%)	189 (30.9)	157 (25.7)	188 (30.8)	534 (29.1)
Hypercholesterolemia, *n* (%)	460 (75.3)	465 (76.1)	483 (79.1)	1408 (76.8)
Hypertension, *n* (%)	530 (86.7)	536 (87.7)	533 (87.2)	1599 (87.2)
Family history of CVD, *n* (%)	146(23.9)	151 (24.7)	154 (25.2)	451 (24.6)
Current smoker,* n* (%)				
Current	111 (18.2)	90 (14.7)	86 (14.1)	287 (15.7)
Former	154 (25.2)	147 (24.1)	153 (25.0)	454 (24.8)
Never	346 (56.6)	374 (61.2)	372 (60.9)	1092 (59.6)
Food and nutrient consumption				
Lentils (g/day)	4 ± 2	6 ± 2	9 ± 6	6 ± 4
Chickpeas (g/day)	3 ± 2	5 ± 2	8 ± 5	5 ± 4
Dry beans (g/day)	3 ± 2	4 ± 2	8 ± 6	5 ± 4
Fresh peas (g/day)	1 ± 2	3 ± 3	6 ± 12	3 ± 7
Total meat (g/day)	139 ± 56	135 ± 48	128 ± 54	134 ± 53
Total fish (g/day)	98 ± 47	103 ± 59	102 ± 48	101 ± 52
Total vegetables (g/day)	307 ± 139	325 ± 136	364 ± 161	332 ± 148
Total fruits (g/day)	353 ± 192	351 ± 184	378 ± 199	361 ± 192
Total cereals (g/day)	233 ± 88	238 ± 75	222 ± 84	231 ± 83
Total dairy (g/day)	368 ± 221	370 ± 202	389 ± 221	375 ± 215
Total olive oil (g/day)	40 ± 16	40 ± 15	38 ± 17	39 ± 16
Total nuts (g/day)	11 ± 14	11 ± 12	11 ± 13	11 ± 13
Total alcohol (g/day)	11 ± 17	9 ± 13	8 ± 13	10 ± 14
Total energy (kcal/day)	2373 ± 587	2189 ± 483	2287 ± 544	2283 ± 545

Tertile ranges (g of legume/day): T1 = [−4.41, 15.2); T2 = [15.23, 22.4], T3 = (22.43, 174.9] at baseline

All dietary variables were adjusted for total energy intake using the residual method

*CVD* cardiovascular disease, *MET* metabolic equivalent of task, *T* Tertiles

Values are means ± standard deviations for continuous variables or number (%) for categorical variables

### Identification of legume-related metabolites

Table 2 presents a summary of the number of selected metabolites and their respective validation with Pearson correlation coefficients between the identified metabolite profile and the amount of legume consumption in both the discovery cohort and the internal validation population (PREDIMED 1-year). A total of 40 metabolites were associated with legume consumption using elastic net regression. Among these, 18 were positively associated with higher consumption of legumes, while 22 were negatively associated with higher consumption of legumes. In addition, Additional file 1: Table S2 shows the correlation between of the legume-related metabolites and each food group or adherence to the MedDiet. Additional file 1: Table S3 provides means and SD for specific metabolites selected 10 times during the tenfold cross-validation elastic net regressions using lambda.min. The Pearson correlations between the identified metabolite profiles and legume consumption at baseline and at 1-year of follow-up were 0.17 (95% CI 0.12, 0.23), and 0.15 (95% CI 0.10, 0.19), respectively.

**Table 2:** Pearson correlation coefficients between metabolomics signatures and legume consumption

	Baseline data				Internal validation population (Year 1 data in PREDIMED)
	Pearson correlation with metabolomic signature (95% CI)	Total No. Metabolites^a^	No. of metabolites with positive coefficients	No. of metabolites with negative coefficients	Pearson correlation (95% CI)
Total legumes, g/d^b^	0.17 (0.12, 0.23)	40	18	22	0.15 (0.10, 0.19)

*CI* confidence interval, *PREDIMED* PREvención con DIeta MEDiterránea

^a^Metabolite coefficients obtained 10 times in the cross-validation procedure for the elastic net continuous approach. Results using the lambda.min option

^b^Adjusted for total energy intake using the residual method

**Fig. 1:**
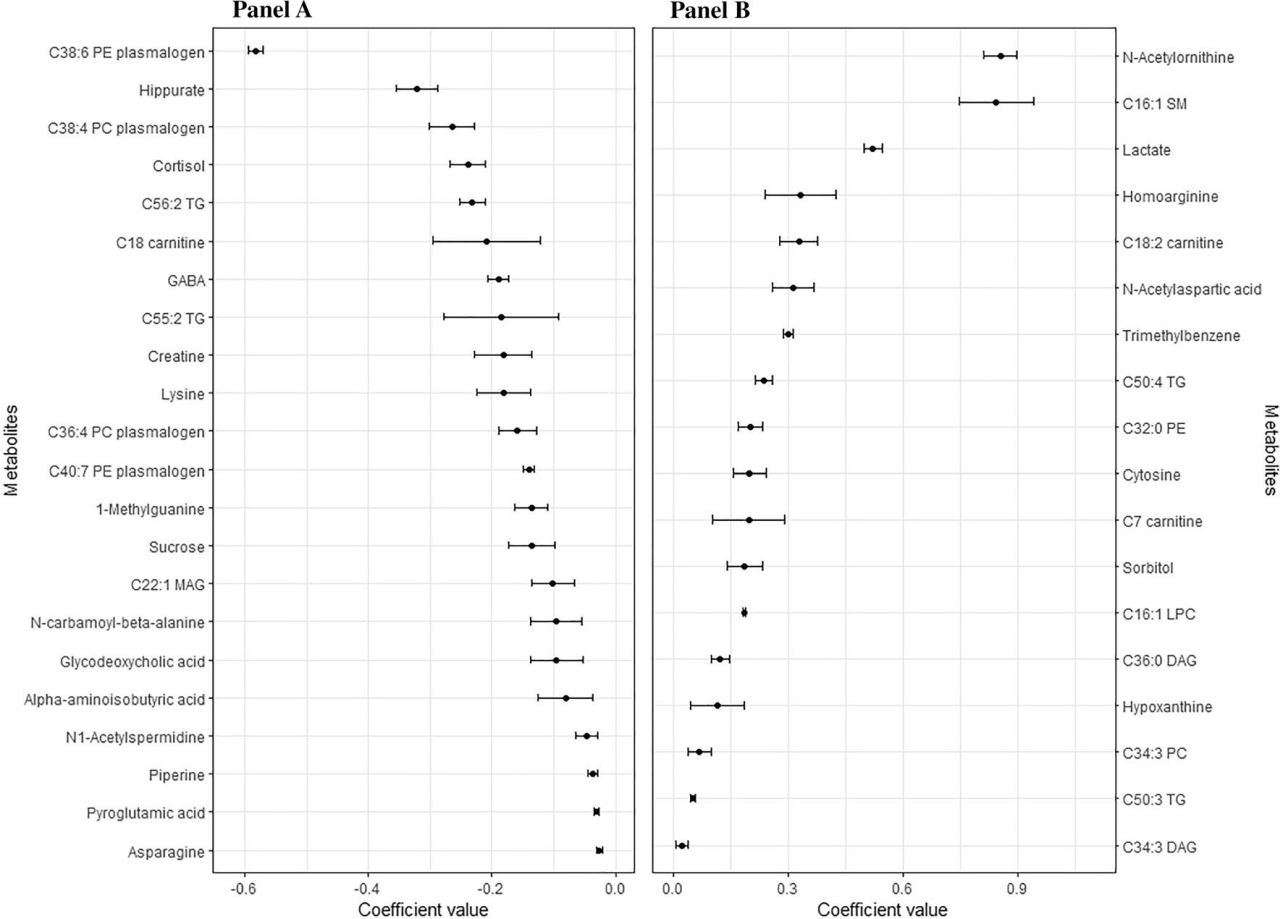
Coefficients for the metabolites selected ten times in the 10-CV elastic net for legume consumption. Mean and SD of the set of the metabolites selected ten times in the ten times iterated tenfold-cross validation of the elastic continuous regression procedure (using lambda.min) employing the whole dataset of subjects (n = 1833). Metabolites with negative coefficients (n = 22) are plotted in **A**, whereas those with positive coefficients (n = 18) are shown in **B**. *DAG* diacylglycerol, *GABA* γ-aminobutyric acid, *LPC* lysophosphatidylcholine, *MAG* monoacylglycerol, *PC* phosphatidylcholine, *PE* phosphatidylethanolamine, *SM* Sphingomyelin, *TG* triacylglycerol

In Fig. 1, the selected metabolites resulting from the tenfold cross-validation of elastic net regressions are presented in the descending order of their coefficient values. The metabolites C38:6 Phosphatidylethanolamine (PE) plasmalogen, hippurate, C38:4 Phosphatidylcholine (PC) plasmalogen, cortisol, C56:2 Triacylglycerol (TG), and C18 carnitine exhibited the most robust negative coefficient values. Conversely, the metabolites N-Acetylornithine, C16:1 Sphingomyelin (SM), lactate, homoarginine, C18:2 carnitine, and N-Acetylaspartic acid displayed the strongest positive coefficients.

### Association of legume-related metabolites with T2D and CVD risk

Additional file 1: Tables S4 and S5 show baseline and 1-year characteristics of the study participants based on case-cohort studies, T2D and CVD respectively. Individuals with incident T2D (Additional file 1: Table S4) were more likely to be men and smokers in comparison with those in the control group. Additionally, T2D cases had a higher BMI, waist circumference, and a greater prevalence of hypertension. Individuals with incident CVD are more likely to be men, current smokers, and have higher waist circumference compared to controls (Additional file 1: Table S5).

After adjusting for lifestyle and dietary risk factors, the hazard ratio (HR) and 95% confident interval (CI) for T2D incidence per SD increment in the metabolite profile model of legume consumption was 0.75 (95% CI 0.61, 0.94; p = 0.01) (comprising 245 cases) at baseline, and 0.99 (95% CI 0.73, 1.33; p = 0.92) at 1-year of follow-up consisting of 161 cases at 1-year (Table 3). No significant differences were observed after adjusting the models for self-reported legume consumption. The metabolite profile of legume consumption did not show a significant association with the incidence of CVD, even after adjusting for potential confounders and self-reported legume consumption (Table 3).

**Table 3:** Legume metabolite profile and risks of type 2 diabetes and cardiovascular disease in PREDIMED study

	PREDIMED baseline^a^		PREDIMED 1 year^b^	
	HR (95% CI)	*p*	HR (95% CI)	*p*
Type 2 diabetes				
*Legume consumption*	*20 g/day*		*22 g/day*	
No. of cases/total participants	245/923		161/704	
Score of metabolites predicting legumes				
Model 1	0.84 (0.69, 1,02)	0.075	1.01 (0.77, 1.33)	0.942
Model 2	0.76 (0.61, 0.94)	0.012	1.00 (0.75, 1.33)	0.997
Model 3	0.75 (0.61, 0.94)	0.012	0.99 (0.73, 1.33)	0.921
Model 4	0.77 (0.62, 0.95)	0.017	0.98 (0.73, 1.32)	0.915
Cardiovascular disease				
*Legume consumption*	*21 g/day*		*11 g/day*	
No. of cases/total participants	222/993		159/916	
Score of metabolites predicting legumes				
Model 1	1.00 (0.89, 1.13)	0.942	1.00 (0.89, 1.13)	0.942
Model 2	1.06 (0.90, 1.23)	0.476	1.01 (0.84, 1.22)	0.915
Model 3	1.03 (0.88, 1.21)	0.697	1.01 (0.84, 1.24)	0.858
Model 4	1.01 (0.86, 1.19)	0.817	1.01 (0.84, 1.23)	0.869

*CI* confidence interval, *HR* hazard ratio, *MET* metabolic equivalent of task, *PREDIMED* PREvención con DIeta MEDiterránea, *BMI* body mass index, *CHD* coronary heart disease, *CVD* cardiovascular disease, *T2D* type 2 diabetes

Model 1: adjusted for age (years), sex, and propensity scores; stratified by intervention group and recruitment center

Model 2: model 1 + BMI, smoking status (never, former, or current smoker), alcohol intake and squared alcohol intake (grams per day), education level (primary, secondary, academic), physical activity (MET-min/day), family history of CHD (yes/no), hypertension (yes/no), hypercholestrolemia (yes/no), use of antihypercholesterolemia medication (yes/no) and use of antihypertensive medication (yes/no)

Model 3: model 2 + , intake of total meat, fish, vegetables, fruits, cereals, dairy, nuts, and olive oil (g/day)

Model 4: model 3 + total legumes (g/day) from which the metabolite set was derived

All dietary variables were adjusted for total energy intake using the residual method

^a^Analysis of CVD risk was performed among the 993 participants of the PREDIMED CVD case–cohort data set or 923 in the T2D case–cohort data set. Cox proportional hazard models, with Barlow weights, were used to estimate HRs and their 95% CIs for CVD or T2D. Person-time of follow-up was estimated as the interval between the baseline date and date of CVD or T2D event, death, or date of the last participant contact, whichever came first. HRs refer to 1-SD increase in correlated multimetabolite score

^b^Legume consumption, metabolic signatures, and covariates were assessed at year 1, and outcome was the incident CVD events occurred after the year 1 visit through the end of follow-up. The analytic models were the same as in the baseline analysis. A total of 916 participants were included in the CVD analyses and 704 in the T2D analyses

In the sensitivity analysis, legume-related metabolites were assessed for their association with T2D and CVD incidence separately for men and women (Additional file 1: Table S6), with similar trends as the main analysis. This analysis was further extended to individuals for both PREDIMED control and Mediterranean intervention groups. The inverse association between the legume metabolomic profile and T2D risk was maintained in those participants in the MedDiet groups although the association was attenuated in the control group. No associations were also shown in case of CVD incidence (Additional file 1: Table S7).

## Discussion

To our knowledge, this is the first study to determine a plasma metabolite profile of legume consumption and to test its association with cardiometabolic diseases, showing a novel dimension in understanding diet-disease relationships. We identified 22 metabolites that were negatively and 18 positively associated with legume consumption, encompassing AAs, and various classes of lipid metabolites including diacylglycerols (DAGs), triacylglycerols, plasmalogens, and sphingomyelins in a sample of individuals participating in the PREDIMED study. The legume-related metabolite profile was shown to be inversely associated with T2D incidence but not with the incidence of CVD, after adjustment for potential confounders and self-reported legume consumption.

The influence of legume consumption on glucose metabolism and T2D risk remains a subject of uncertainty [35–37]. A meta-analysis of RCTs reported that legume consumption contributed to the reductions in plasma glucose levels, Homeostatic Model Assessment of Insulin Resistance (HOMA-IR), total (TC) and Low Density Lipoprotein (LDL-C) cholesterol, and several other recognized risk factors for T2D and CVD [35]. A recent systematic review and meta-analysis of 10 prospective studies investigating the associations between total legume consumption and T2D (high vs. low), reported a nonsignificant association [35]. This lack of significance may be attributed to the inherent heterogeneity across the included studies. Nevertheless, the protective effects of legume consumption against T2D have primarily been observed in populations adhering to a MedDiet, a dietary pattern that has been consistently associated with a reduced risk of T2D [35, 36].

With regard to CVD, previous systematic reviews and meta-analyses have consistently reported an inverse association between legume consumption and CVD risk [7, 9]. However, the role that legume consumption has in the risk of stroke remains controversial, mainly because stroke includes a heterogeneous group of diseases with a wide range of aetiologies. For example, in the PREDIMED population a positive association between legume consumption and stroke was reported [38].

Despite the evidence for legume consumption in lowering cardiometabolic risks and the complex matrix of compounds of the *Leguminosae* family (including complex carbohydrates, sugars, AAs, TGs, phosphatidylcholine, and sphingolipids among others) [39], the associations between identified plasma metabolites signatures of legume consumption and chronic diseases remains sparse [40, 41]. Mainly because of the limited evidence on legumes consumption metabolomic signature and the classification of legumes done in previous studies [42]. In this regard, our findings extend current understanding in this area by identifying potential mechanistic pathways involved in the associations between legume consumption, especially for lentils, chickpeas and dry beans, and cardiometabolic risks especially related to T2D.

In our study, N-acetylornithine and homoarginine were positively associated with legume consumption. These metabolites have also been associated with the consumption of vegetables and plant-based diets rich in legumes [43, 44], both with recognized cardiometabolic benefits [45], especially in relation to glucose metabolism. N-acetylornithine plays a role in arginine synthesis [43, 46], and homoarginine is related to the metabolism of arginine and lysine [47]. Arginine has previously been linked with enhanced insulin secretion and sensitivity [48]. The unexpected negative association reported between lysine and legume consumption, when legumes are considered an important source of lysine [41] might be explained by a metabolic alternative pathway for homoarginine synthesis that involves the use of lysine instead of ornithine in the urea-cycle enzymes [49, 50], resulting in a possible up-regulation of homoarginine.

Our legume-related metabolite profile includes several lipids positively related to legume consumption, such as C34:3 PC and C16:1 lysophosphatidylcholine (LPC). Only C16:1 LPC has been previously related to legumes [39] and none of these metabolites have been previously linked to T2D. To our knowledge, it is the first time that negative association between the C36:4 PC metabolite and T2D risk was also reported. The number of carbons and position of bounds of lipids plays a crucial role in T2D. For instance, TGs or DAGs with high number of carbons and > 2 double bonds have been inversely associated with T2D, with carbon chain length being more influential than the number of double bonds [51–54]. In our study C50:4 TG, C50:3 TG, C34:3 DAG, and C36:0 DAG were positively associated with legume consumption. However, the specific effect of these lipid species on T2D remains uncertain. In our study C50:3 TG and C34:3 DAG metabolites showed a positive association with T2D risk (data not shown). In a previous report, C50:3 TG was also associated with an increased risk of diabetes, whereas C34:3 DAG was not [51], therefore further studies are needed to clarify these controversial findings. Other lipids, as sphingolipid C16:1 SM, were also included in the legume-related-metabolite signature and showed a decreased risk association for T2D. Biological pathway analysis in combination with genetics and mice experiments indicates that the downregulation or upregulation of sphingolipid is a causal factor in early-stage T2D pathophysiology and is associated with a decreased risk of T2D [55, 56]. The positive association between C7 and C18:2 acylcarnitines and legume consumption could be an indirect reflection of high fish consumption by those individuals allocated in the highest tertile of legume consumption, as fish has been established as one of the main dietary sources of acylcarnitines, although no association between short chain acylcarnitines and diabetes risk has been previously reported [57].

We observed that glycodeoxycholic acid, a derivative of deoxycholic acid and glycine, was inversely related to legume consumption and higher levels of this metabolite have been reported in individuals with T2D and has been associated to an increased risk of T2D [58, 59].

We also found that the neurotransmitter gamma-aminobutyric acid (GABA) was negatively related to legume consumption. In this regard, high GABA levels have been previously reported in individuals with T2D and with high fasting glucose levels [60]. Similarly, we observed a negative association between cortisol and legume consumption is noteworthy, as alterations in the diurnal cortisol rhythm and elevated morning serum cortisol levels have been related to adiposity, dyslipidemia, alterations in glucose metabolism, and an increased predisposition to T2D and CVD [61].

We observed a negative correlation between legume consumption and several organic acids such as N1-Acetylspermidine, alpha-aminoisobutyric acid (AABA), creatine and pyroglutamic acid (PA). This correlation may arise from AAs metabolism. PA is produced during peptide bond formation between the gamma-carboxyl group and alpha-amino group of glutamic acid [62]; creatine is naturally synthesized within the human body from the AAs glycine, arginine and methionine [63], although it can also be obtained through dietary sources; AABA is a non-proteinogenic AA formed as a by-product of either cysteine biosynthesis or the metabolic pathways of methionine, threonine, serine, and glycine [64]; and N1-Acetylspermidine is synthesized through the enzymatic action of spermidine/spermine N1-acetyltransferase [65]. Legumes are rich in AAs (such as asparagine, arginine, glutamic acid, and others) that can alter the metabolic pathway of these metabolites.

Our analysis showed that our metabolic score is associated with a 23% reduction in the risk of developing T2D. Nevertheless, this association was attenuated and non-significant when participants with incident diabetes during the first year of follow-up were excluded probably because of the effect of the intervention and lower statistical power. In addition, this signature did not show statistically significant associations with CVD risk. The inverse association between the legume metabolomic profile and T2D incidence observed especially in those participants in the Mediterranean groups may be explained by the beneficial effect may have the interventions on the risk of diabetes.

We acknowledge some limitations to this analysis. Firstly, owing to the cross-sectional design, the establishment of causal relationships cannot be inferred. Secondly, notwithstanding the utilization of a metabolic analysis including an extensive spectrum of metabolites, it is noteworthy that certain metabolites with established correlations to legume consumption were neither included within the model's selection nor discerned within the platforms employed for our study. Thirdly, distinguishing metabolites originating directly from diet and those from induced metabolic pathways is challenging. These selected metabolites cannot be considered definitive biomarkers of legume consumption because endogenous synthesis secondary to its consumption and related lifestyle factors of legume consumers may be responsible for some of the identified metabolites found. Fourthly, the presence of residual effects of pathologies on the results cannot be excluded. However, given the low incidence of these pathologies in the first year (147 out of 1837, less than 10%), the impact on the results is not substantial. Finally, participants were elderly Mediterranean individuals with elevated CVD risk, who consumed relatively small amounts of legumes daily. This demographic and dietary context raises considerations about the broader applicability of our results to different age ranges or other populations. Regarding strengths, we have to mention the large sample size studied, detailed covariate data used to control for confounding, the inclusion of > 300 known metabolites in the agnostic matching learning models implemented, and the internal validation of the outcomes executed within the discovery cohort, employing baseline data.

## Conclusion

In this analysis, a set of metabolites exhibited significant associations with legume consumption, and the scores derived from these identified metabolites showed an inverse association with the risk of T2D incidence in an elderly Mediterranean population at high risk of CVD. However, this metabolite profile was not associated with the risk of CVD.

Our findings provide a potential objective measurement of legume consumption at population level based on a set of plasma metabolites which might reduce the assessment errors inherent to FFQ’s; and the most relevant, is that the identified metabolomic signature related to legume consumption might contribute to the understanding the metabolic pathways induced by the consumption of legumes in relation to the pathophysiology of diabetes and CVD, potentially contributing to the development of personalised nutritional strategies in the prevention and management of non-communicable chronic diseases such as T2D and CVD. However, further investigations are warranted in the future to establish metabolite signatures that accurately capture the metabolic responses induced by direct consumption of this food group. At the same time, a comprehensive understanding of the metabolic pathways involved in the progression to T2D and CVD after legume consumption is essential.

### Supplementary Information

**Additional file 1: Figure S1.** Diagram of participant inclusion and analytical methodology. **Table S1.** Participant’s characteristics according to tertiles of energy-adjusted total legume consumption at one year of follow-up. **Table S2.** Correlations between the legume-related metabolites and other food groups and Mediterranean diet adherence. **Table S3.** Mean and SD metabolite coefficients associated to legume consumption. **Table S4.** Participant’s general characteristics according to T2D case-cohort database. **Table S5.** Participant’s general characteristics according to CVD case-cohort database. **Table S6.** Legume metabolite profile and risks of type 2 diabetes and cardiovascular disease by sex. **Table S7.** Legume metabolite profile and risk of type 2 diabetes and cardiovascular disease by intervention groups.

## Data Availability

The dataset generated and/or analyzed during the current study are not publicly available due the lack of authorization from PREDIMED participants. Requestors wishing to access the PREDIMED trial data used in this study can make a request to the corresponding author and it will then be passed to members of the PREDIMED Steering Committee for deliberating.

## Abbreviations

AA: Amino acid
AABA: Alpha-aminoisobutyric acid
GABA: Aminobutyric acid
BMI: Body mass index
CVD: Cardiovascular disease
CI: Confidence interval
CHD: Coronary heart disease
CV: Cross-validation
DAGs: Diacylglycerols
FFQs: Food frequency questionnaires
HbA1c: Glycated hemoglobin A1c
HR: Hazard ratio
HOMA-IR: Homeostatic Model Assessment of Insulin Resistance
LC–MS/MS: Liquid chromatography-tandem mass spectrometry
LPC: Lysophosphatidylcholine
MSE: Mean square error
MedDiet: Mediterranean diet
OGTT: Oral glucose tolerance test
PC: Phosphatidylcholine
PE: Phosphatidylethanolamine
PREDIMED: Prevención con dieta mediterránea
PA: Pyroglutamic acid
RCT: Randomized controlled trials
SM: Sphingomyelin
SD: Standard deviation
SE: Standard error
TC: Total cholesterol
TG: Triacylglycerol
T2D: Type 2 diabetes
