# Supplementary material for: Plasma metabolite profile of legume consumption and future risk of type 2 diabetes and cardiovascular disease
Source: Cardiovasc Diabetol. 2024 Jan 20;23:38. doi: 10.1186/s12933-023-02111-z (PMC10800064; doi:10.1186/s12933-023-02111-z)
Supplement: Supplementary file 1 — Additional file 1: Figure S1. Diagram of participant inclusion and analytical methodology. Table S1. Participant’s characteristics according to tertiles of energy-adjusted total legume consumption at one year of follow-up. Table S2. Correlations between the legume-related metabolites and other food groups and Mediterranean diet adherence. Table S3. Mean and SD metabolite coefficients associated to legume consumption. Table S4. Participant’s general characteristics according to T2D case-cohort database. Table S5. Participant’s general characteristics according to CVD case-cohort database. Table S6. Legume metabolite profile and risks of type 2 diabetes and cardiovascular disease by sex. Table S7. Legume metabolite profile and risk of type 2 diabetes and cardiovascular disease by intervention groups. [file 12933_2023_2111_MOESM1_ESM.docx]

**Additional figure 1.** Diagram of participant inclusion and analytical methodology

**Predimed Trial Participants**;

N = 7447

Baseline Metabolomics population after merging;

n = 1882

**Exclusion for (n = 49);**

No FFQ; n = 11

Unlikely energy intake; n = 34

Missing metabolites; n = 4

**Exclusion for lack of metabolic data:**

n = 5565

PREDIMED-CVD subcohort Project;

n = 989

PREDIMED-T2D subcohort Project;

n = 892

T2D-OGTT Project;

n = 130

**Duplicates;** n = 122

10-fold CV ENR using the whole discovery population

10-fold CV ENR approaching splitting into training (90%) and validation (10%)

Model performance correlation between metabolite signature and self-reported food consumption in the discovery population

**Exclusion for (n = 312):**

No metabolite data;

n = 58

No FFQ at one year;

n =254

**Validation population**;

n = 1521

Metabolomics Signature

**Internal validation:** correlation between metabolite signature and self-reported food consumption in Validation population

PREDIMED T2D subcohort

PREDIMED CVD subcohort

**Discovery Population;**

n = 1833

Abbreviation: FFQ, food frequency questionnaire; CV, cross-validation; ENR, elastic net regression.

| **Additional table 1. Participant’s characteristics according to tertiles of energy-adjusted total legume consumption at one year of follow-up^1^** | | | | | | |
| --- | --- | --- | --- | --- | --- | --- |
|  |  |  |  |  |  |  |
| **Characteristics** | | **T1**  **(*n* = 507)** | **T2**  **(*n* = 507)** | **T3**  **(*n* = 507)** | | **Overall**  **(*n* = 1521)** |
| **Total legume consumption (g/day)** | | 13 ± 4 | 21 ± 2 | 33 ± 11 | | 22 ± 11 |
| **Age (years)** | | 67 ± 6 | 67 ± 6 | 68 ± 6 | | 67 ± 6 |
| **Sex, women, *n* (%)** | | 305 (60.2) | 291 (57.4) | 278 (54.8) | | 874 (57.5) |
| **Education level, *n*(%)** | |  |  |  | |  |
| Primary | | 373 (73.6) | 368 (72.6) | 382 (75.3) | | 1123 (73.8) |
| Secondary | | 90 (17.8) | 93 (18.3) | 71 (14.0) | | 254 (16.7) |
| Academic | | 34 (6.7) | 33 (6.5) | 49 (9.7) | | 116 (7.6) |
| **Body mass index (kg/m^2^)** | | 30.0 ± 3.7 | 29.7 ± 3.3 | 29.8 ± 3.6 | | 29.8 ± 3.6 |
| **Waist circumference (cm)** | | 100 ± 10 | 100 ± 10 | 100 ± 11 | | 100 ± 10 |
| **Leisure time physical activity  level (MET min/day)** | | 232 ± 231 | 256 ± 229 | 262 ± 252 | | 250 ± 238 |
| **Type 2 diabetes** | | 148 (29.2) | 153 (30.2) | 162 (32.0) | | 463 (30.4) |
| **Hypercholesterolemia, *n*(%)** | | 379 (74.8) | 369 (72.8) | 404 (79.7) | | 1152 (75.7) |
| **Hypertension, *n*(%)** | | 447 (88.2) | 435 (85.8) | 435 (85.8) | | 1317 (86.6) |
| **Family history of CVD, *n*(%)** | | 125 (24.7) | 133 (26.2) | 120 (23.7) | | 378 (24.9) |
| **Smoking, *n* (%)** | |  |  |  | |  |
| Current | | 93 (18.3) | 77 (15.2) | 66 (13.0) | | 236 (15.5) |
| Former | | 115 (22.7) | 122 (24.1) | 144 (28.4) | | 381 (25.0) |
| Never | | 299 (59.0) | 308 (60.7) | 297 (58.6) | | 904 (59.4) |
| **Food and nutrient consumption** | |  |  |  | |  |
| **Lentiles (g/day)** | | 4 ± 2 | 7 ± 2 | 10 ± 6 | | 7 ± 5 |
| **Chickpeas (g/day)** | | 3 ± 2 | 6 ± 3 | 9 ± 5 | | 6 ± 4 |
| **Dry beans (g/day)** | | 3 ± 2 | 5 ± 3 | 9 ± 6 | | 6 ± 5 |
| **Fresh peas (g/day)** | | 2 ± 2 | 3 ± 3 | 5 ± 5 | | 3 ± 4 |
| **Total meat (g/day)** | | 129 ± 50 | 125 ± 45 | 118 ± 48 | | 124 ± 48 |
| **Total fish (g/day)** | | 104 ± 45 | 106 ± 40 | 108 ± 45 | | 106 ± 43 |
| **Total vegetables (g/day)** | | 327 ± 136 | 344 ± 128 | 367 ± 135 | | 346 ± 134 |
| **Total fruits (g/day)** | | 369 ± 191 | 396 ± 176 | 404 ± 197 | | 390 ± 189 |
| **Total cereals (g/day)** | | 232 ± 77 | 227 ± 74 | 216 ± 77 | | 225 ± 76 |
| **Total dairy (g/day)** | | 360 ± 202 | 358 ± 198 | 379 ± 228 | | 366 ± 210 |
| **Total olive oil (g/day)** | | 45 ± 16 | 45 ± 16 | 44 ± 16 | | 45 ± 16 |
| **Total nuts (g/day)** | | 20 ± 21 | 21 ± 21 | 23 ± 23 | | 21 ± 22 |
| **Total alcohol (g/day)** | | 10 ± 14 | 8 ± 11 | 8 ± 12 | | 9 ± 12 |
| **Total energy (kcal/day)** | | 2320 ± 581 | 2260 ± 551 | 2276 ± 498 | | 2286 ± 545 |
| Abbreviations: CVD, cardiovascular disease; MET, metabolic equivalent of task;  ^1^Values are means ± standard deviations for continuous variables or number (%) for categorical variables.  T: Tertiles. Tertile ranges (g of legume/day): T1 = (-1.36, 17.3); T2 = (17.34, 24.6), T3 = (24.60, 86.8) at first year of follow-up.  All dietary variables were adjusted for total energy intake using the residual method. | | | | | | |
|  |  |  |  |  |  |  |
|  |  |  |  |  |  |  |
|  |  |  |  |  |  |  |
| **Additional table 2. Correlations between the legume-related metabolites and other food groups and Mediterranean diet adherence** | | | | | | |
|  | **Pearson correlation (95% CI) at baseline** | | | **Pearson correlation (95% CI) at 1 year** | | |
| **Total meat (g/day)^1^** | -0.18 (-0.22, -0.13) | | | -0.17 (-0.22, -0.12) | | |
| **Total fish (g/day) ^1^** | -0.07 (0.11, -0.02) | | | -0.05 (-0.99, 0.00) | | |
| **Total vegetables (g/day) ^1^** | 0.08 (0.03, 0.12) | | | 0.07 (0.02, 0.12) | | |
| **Total fruits (g/day) ^1^** | -0.01 (-0.05, 0.04) | | | 0.02 (-0.03, 0.07) | | |
| **Total cereals (g/day) ^1^** | -0.07 (-0.12, -0.02) | | | -0.07 (-0.12, -0.02) | | |
| **Total dairy (g/day) ^1^** | 0.12 (0.07, 0.16) | | | 0.06 (0.01, 0.11) | | |
| **Total olive oil (g/day) ^1^** | -0.09 (-0.13, -0.04) | | | -0.07 (-0.12, -0.02) | | |
| **Total nuts (g/day) ^1^** | 0.02 (-0.02, 0.07) | | | 0.04 (-0.01, 0.09) | | |
| **Total alcohol (g/day) ^1^** | -0.10 (-0.14, -0.05) | | | -0.12 (-0.17, -0.07) | | |
| **Mediterranean diet** | -0.01 (-0.05, 0.04) | | | -0.01 (-0.06, 0.04) | | |
| Abbreviations: CI, confidence interval.  1. Adjusted for total energy intake using the residual method. | | | |  | | |

| **Additional table 3. Mean and SD metabolite coefficients associated to legume consumption** | | |
| --- | --- | --- |
| **Names** | **Mean** | **SD** |
| Alpha-aminoisobutyric acid | -0,081 | 0,045 |
| Asparagine | -0,026 | 0,005 |
| C16:1 LPC | 0,185 | 0,004 |
| C16:1 SM | 0,844 | 0,097 |
| C18:2 carnitine | 0,328 | 0,049 |
| C18 carnitine | -0,209 | 0,088 |
| C22:1 MAG | -0,101 | 0,035 |
| C32:0 PE | 0,202 | 0,032 |
| C34:3 DAG | 0,023 | 0,015 |
| C34:3 PC | 0,068 | 0,031 |
| C36:0 DAG | 0,123 | 0,024 |
| C36:4 PC plasmalogen | -0,159 | 0,031 |
| C38:4 PC plasmalogen | -0,265 | 0,036 |
| C38:6 PE plasmalogen | -0,582 | 0,012 |
| C40:7 PE plasmalogen | -0,140 | 0,009 |
| C50:3 TG | 0,051 | 0,008 |
| C50:4 TG | 0,237 | 0,022 |
| C55:2 TG | -0,185 | 0,093 |
| C56:2 TG | -0,232 | 0,020 |
| C7 carnitine | 0,197 | 0,094 |
| Cortisol | -0,239 | 0,029 |
| Creatine | -0,182 | 0,047 |
| Cytosine | 0,199 | 0,044 |
| GABA | -0,190 | 0,017 |
| Glycodeoxycholic acid | -0,095 | 0,042 |
| Hippurate | -0,321 | 0,033 |
| Homoarginine | 0,333 | 0,092 |
| Hypoxanthine | 0,115 | 0,070 |
| Lactate | 0,521 | 0,023 |
| Lysine | -0,181 | 0,044 |
| 1-Methylguanine | -0,136 | 0,027 |
| N-Acetylaspartic acid | 0,312 | 0,054 |
| N-Acetylornithine | 0,854 | 0,043 |
| N1-Acetylspermidine | -0,046 | 0,018 |
| N-carbamoyl-beta-alanine | -0,096 | 0,041 |
| Piperine | -0,037 | 0,008 |
| Pyroglutamic acid | -0,030 | 0,004 |
| Sorbitol | 0,186 | 0,046 |
| Sucrose | -0,135 | 0,038 |
| LPC | 0,301 | 0,013 |
| Abbreviations: DAG: diacylglycerol; GABA: γ-aminobutyric acid; LPC: lysophosphatidylcholine; MAG: monoacylglycerol; PC: phosphatidylcholine; PE: phosphatidylethanolamine; SD: standard deviation; SM: Sphingomyelin; TG: triacylglycerol. | | |

| **Additional table 4. Participant’s general characteristics according to T2D case-cohort database^1^** | | | | | | | |
| --- | --- | --- | --- | --- | --- | --- | --- |
|  |  | **Baseline Visit** | | | **1 year** | | |
| **Characteristics** |  | **Control**  **(*n* = 678)** | **Case**  **(*n* = 245)** | **Overall**  **(*n* = 923)** | **Control**  **(*n* = 543)** | **Case**  **(*n* = 161)** | **Overall**  **(*n* = 704)** |
| **Age (years)** |  | 67 ± 6 | 66 ± 6 | 67 ± 6 | 67 ± 6 | 66 ± 5 | 67 ± 6 |
| **Sex, women, *n* (%)** |  | 428 (63.1) | 135 (55.1) | 563 (61.0) | 349 (64.3) | 84 (52.2) | 433 (61.5) |
| **Education level, *n*(%)** |  |  |  |  |  |  |  |
| Primary |  | 493 (72.7) | 187 (76.3) | 680 (73.7) | 395 (72.7) | 123 (76.4) | 518 (73.6) |
| Secondary |  | 118 (17.4) | 38 (15.5) | 156 (16.9) | 95 (17.5) | 25 (15.5) | 120 (17.0) |
| Academic |  | 49 (7.2) | 19 (7.8) | 68 (7.4) | 40 (7.4) | 12 (7.5) | 52 (7.4) |
| **Body mass index (kg/m^2^)** |  | 29.9 ± 3.6 | 30.9 ± 3.3 | 30.1 ± 3.6 | 29.7 ± 3.5 | 30.7 ± 3.2 | 29.9 ± 3.5 |
| **Waist circumference (cm)** |  | 100 ± 11 | 103 ± 10 | 101 ± 11 | 99 ± 11 | 103 ± 10 | 100 ± 11 |
| **Leisure time physical activity level (MET min/day)** |  | 236 ± 232 | 246 ± 231 | 239 ± 232 | 242 ± 235 | 257 ± 226 | 245 ± 233 |
| **Hypercholesterolemia, *n*(%)** |  | 577 (85.1) | 195 (79.6) | 772 (83.6) | 457 (84.2) | 127 (78.9) | 584 (83.0) |
| **Hypertension, *n*(%)** |  | 615 (90.7) | 235 (95.9) | 850 (92.1) | 494 (91.0) | 155 (96.3) | 649 (92.2) |
| **Family history of CVD, *n*(%)** |  | 199 (29.4) | 55 (22.4) | 254 (27.5) | 161 (29.7) | 41 (25.5) | 202 (28.7) |
| **Smoking, *n* (%)** |  |  |  |  |  |  |  |
| Current |  | 110 (16.2) | 61 (24.9) | 171 (18.5) | 87 (16.0) | 40 (24.8) | 127 (18.0) |
| Former |  | 152 (22.4) | 55 (22.4) | 207 (22.4) | 123 (22.7) | 39 (24.2) | 162 (23.0) |
| Never |  | 416 (61.4) | 129 (52.7) | 545 (59.0) | 333 (61.3) | 82 (50.9) | 415 (58.9) |
| **Medication use, *n* (%)** |  |  |  |  |  |  |  |
| Lipid-lowering drugs |  | 331 (48.8) | 125 (51.0) | 456 (49.4) | 262 (48.3) | 84 (52.2) | 346 (49.1) |
| Antihypertensive drugs |  | 514 (75.8) | 204 (83.3) | 718 (77.8) | 419 (77.2) | 131 (81.4) | 550 (78.1) |
| **Food and nutrient consumption** |  |  |  |  |  |  |  |
| **Total legumes (g/day)** |  | 21 ± 13 | 18 ± 9 | 20 ± 12 | 22 ± 12 | 21 ± 9 | 22 ± 11 |
| **Total meat (g/day)** |  | 132 ± 49 | 134 ± 47 | 132 ± 49 | 124 ± 47 | 127 ± 45 | 124 ± 46 |
| **Total fish (g/day)** |  | 101 ± 47 | 96 ± 41 | 100 ± 46 | 106 ± 43 | 103 ± 36 | 106 ± 42 |
| **Total vegetables (g/day)** |  | 332 ± 140 | 294 ± 112 | 322 ± 134 | 351 ± 123 | 324 ± 119 | 345 ± 122 |
| **Total fruits (g/day)** |  | 359 ± 188 | 335 ± 174 | 353 ± 184 | 408 ± 188 | 382 ± 181 | 402 ± 186 |
| **Total cereals (g/day)** |  | 228 ± 84 | 237 ± 81 | 231 ± 84 | 216 ± 75 | 236 ± 85 | 221 ± 78 |
| **Total dairy (g/day)** |  | 372 ± 215 | 356 ± 225 | 368 ± 218 | 361 ± 203 | 354 ± 239 | 359 ± 211 |
| **Total olive oil (g/day)** |  | 38 ± 14 | 39 ± 15 | 38 ± 15 | 43 ± 16 | 44 ± 16 | 43 ± 16 |
| **Total nuts (g/day)** |  | 11 ± 13 | 12 ± 14 | 11 ± 13 | 23 ± 24 | 19 ± 17 | 22 ± 22 |
| **Total alcohol (g/day)** |  | 10 ± 15 | 13 ± 18 | 11 ± 16 | 9 ± 11 | 11 ± 15 | 9 ± 12 |
| **Total energy (kcal/day)** |  | 2247 ± 515 | 2297 ± 561 | 2260 ± 527 | 2240 ± 552 | 2271 ± 564 | 2247 ± 555 |
| Abbreviations: T2D, type 2 diabetes; CVD, cardiovascular disease; MET, metabolic equivalent of task.  ^1^Values are means ± standard deviations for continuous variables or number (%) for categorical variables.  All dietary variables were adjusted for total energy intake using the residual method. | | | | | | | |

| **Additional table 5. Participant’s general characteristics according to CVD case-cohort database^1^** | | | | | | |
| --- | --- | --- | --- | --- | --- | --- |
|  | **Baseline Visit** | | | **1 year** | | |
| **Characteristics** | **Control**  **(*n* = 771)** | **Case**  **(*n* = 222)** | **Overall**  **(*n* = 993)** | **Control**  **(*n* = 757)** | **Case**  **(*n* = 159)** | **Overall**  **(*n* = 916)** |
| **Age (years)** | 67 ± 6 | 69 ± 6 | 68 ± 6 | 67 ± 6 | 69 ± 7 | 68 ± 6 |
| **Sex, women, *n* (%)** | 441 (57.2) | 87 (39.2) | 528 (53.2) | 432 (57.1) | 63 (39.6) | 495 (54.0) |
| **Education level, *n*(%)** |  |  |  |  |  |  |
| Primary | 571 (74.1) | 177 (79.7) | 748 (75.3) | 559 (73.8) | 127 (79.9) | 686 (74.9) |
| Secondary | 125 (16.2) | 27 (12.2) | 152 (15.3) | 123 (16.2) | 19 (11.9) | 142 (15.5) |
| Academic | 59 (7.7) | 15 (6.8) | 74 (7.5) | 59 (7.8) | 11 (6.9) | 70 (7.6) |
| **Body mass index (kg/m^2^)** | 29.7 ± 3.6 | 29.6 ± 3.8 | 29.7 ± 3.6 | 29.7 ± 3.6 | 29.7 ± 3.8 | 29.7 ± 3.6 |
| **Waist circumference (cm)** | 100 ± 10 | 102 ± 11 | 100 ± 10 | 100 ± 10 | 102 ± 10 | 100 ± 10 |
| **Leisure time physical activity  level (MET min/day)** | 259 ± 259 | 238 ± 240 | 254 ± 255 | 260 ± 260 | 244 ± 242 | 257 ± 257 |
| **Hypercholesterolemia, *n*(%)** | 565 (73.3) | 130 (58.6) | 695 (70.0) | 552 (72.9) | 91 (57.2) | 643 (70.2) |
| **Hypertension, *n*(%)** | 644 (83.5) | 182 (82.0) | 826 (83.2) | 632 (83.5) | 130 (81.8) | 762 (83.2) |
| **Family history of CVD, *n*(%)** | 191 (24.8) | 42 (18.9) | 233 (23.5) | 187 (24.7) | 29 (18.2) | 216 (23.6) |
| **Smoking, *n* (%)** |  |  |  |  |  |  |
| Current | 94 (12.2) | 46 (20.7) | 140 (14.1) | 94 (12.4) | 35 (22.0) | 129 (14.1) |
| Former | 196 (25.4) | 77 (34.7) | 273 (27.5) | 192 (25.4) | 55 (34.6) | 247 (27.0) |
| Never | 481 (62.4) | 99 (44.6) | 580 (58.4) | 471 (62.2) | 69 (43.4) | 540 (59.0) |
| **Medication use, *n* (%)** |  |  |  |  |  |  |
| Lipid-lowering drugs | 374 (48.5) | 87 (39.2) | 461 (46.4) | 362 (47.8) | 59 (37.1) | 421 (46.0) |
| Antihypertensive drugs | 578 (75.0) | 165 (74.3) | 743 (74.8) | 567 (74.9) | 119 (74.8) | 686 (74.9) |
| **Food and nutrient consumption** |  |  |  |  |  |  |
| **Total legumes (g/day)** | 20 ± 13 | 21 ± 12 | 21 ± 13 | 11 ± 10 | 12 ± 11 | 11 ± 11 |
| **Total meat (g/day)** | 135 ± 55 | 138 ± 64 | 136 ± 57 | 52 ± 50 | 61 ± 47 | 54 ± 49 |
| **Total fish (g/day)** | 106 ± 58 | 96 ± 48 | 104 ± 56 | 44 ± 45 | 38 ± 45 | 43 ± 45 |
| **Total vegetables (g/day)** | 350 ± 158 | 320 ± 165 | 343 ± 160 | 246 ± 139 | 244 ± 154 | 245 ± 142 |
| **Total fruits (g/day)** | 385 ± 202 | 351 ± 200 | 377 ± 202 | 216 ± 188 | 190 ± 198 | 211 ± 190 |
| **Total cereals (g/day)** | 231 ± 82 | 234 ± 89 | 232 ± 84 | 33 ± 74 | 32 ± 79 | 33 ± 75 |
| **Total dairy (g/day)** | 385 ± 218 | 388 ± 219 | 386 ± 218 | 202 ± 211 | 195 ± 222 | 201 ± 213 |
| **Total olive oil (g/day)** | 40 ± 17 | 38 ± 17 | 40 ± 17 | 20 ± 16 | 17 ± 17 | 20 ± 16 |
| **Total nuts (g/day)** | 11 ± 13 | 9 ± 12 | 11 ± 13 | -8 ± 20 | -8 ± 23 | -8 ± 21 |
| **Total alcohol (g/day)** | 9 ± 14 | 10 ± 17 | 9 ± 14 | -11 ± 12 | -7 ± 17 | -10 ± 13 |
| **Total energy (kcal/day)** | 2303 ± 566 | 2329 ± 616 | 2309 ± 577 | 2280 ± 535 | 2354 ± 633 | 2293 ± 553 |
| Abbreviations: CVD, cardiovascular disease; MET, metabolic equivalent of task.  ^1^Values are means ± standard deviations for continuous variables or number (%) for categorical variables.  All dietary variables were adjusted for total energy intake using the residual method. | | | | | | |

| **Additional table 6.** Legume metabolite profile and risks of type 2 diabetes and cardiovascular disease by sex | | | | | | | | | | | |
| --- | --- | --- | --- | --- | --- | --- | --- | --- | --- | --- | --- |
|  |  | **Men** | | | | | **Women** | | | | |
|  |  | **PREDIMED**  **Baseline^a^** | |  | **PREDIMED**  **1 year^b^** | | **PREDIMED**  **Baseline^a^** | |  | **PREDIMED**  **1 year^b^** | |
|  |  | **HR (95% CI)** | ***p*** |  | **HR (95% CI)** | ***p*** | **HR (95% CI)** | ***p*** |  | **HR (95% CI)** | ***p*** |
| **Type 2 diabetes** | |  |  |  |  |  |  |  |  |  |  |
|  | Legume consumption | *19 g/day* |  |  | 22 *g/day* |  | *20 g/day* |  |  | 21 *g/day* |  |
|  | No. of cases/total participants | **110/360** |  |  | **77/271** |  | **135/563** |  |  | **84/433** |  |
|  | Score of metabolites predicting legumes |  |  |  |  |  |  |  |  |  |  |
|  | Model 1 | 0.79 (0.59. 1.05) | 0.103 |  | 1.09 (0.91. 1.31) | 0.344 | 0.90 (0.69. 1.18) | 0.448 |  | 0.96 (0.80. 1.15) | 0.672 |
|  | Model 2 | 0.67 (0.47. 0.95) | 0.025 |  | 1.04 (0.86. 1.26) | 0.684 | 0.82 (0.61. 1.11) | 0.2 |  | 0.93 (0.73. 1.17) | 0.509 |
|  | Model 3 | 0.65 (0.45. 0.93) | 0.019 |  | 1.05 (0.86. 1.29) | 0.624 | 0.86 (0.63. 1.17) | 0.335 |  | 0.93 (0.73. 1.17) | 0.519 |
|  | Model 4 | 0.66 (0.46. 0.95) | 0.025 |  | 1.04 (0.85. 1.28) | 0.681 | 0.86 (0.63. 1.18) | 0.35 |  | 0.94 (0.75. 1.18) | 0.601 |
| **Cardiovascular disease** | |  |  |  |  |  |  |  |  |  |  |
|  | Legume consumption | *20 g/day* |  |  | *11* g/day |  | *20 g/day* |  |  | *10 g/day* |  |
|  | No. of cases/total participants | **135/465** |  |  | **96/421** |  | **87/528** |  |  | **63/495** |  |
|  | Score of metabolites predicting legumes |  |  |  |  |  |  |  |  |  |  |
|  | Model 1 | 1.00 (0.89. 1.13) | 0.942 |  | 1.00 (0.89. 1.13) | 0.942 | 1.00 (0.89. 1.13) | 0.942 |  | 1.00 (0.89. 1.13) | 0.942 |
|  | Model 2 | 1.07 (0.87. 1.32) | 0.505 |  | 0.97 (0.75. 1.26) | 0.807 | 1.01 (0.78. 1.32) | 0.919 |  | 1.04 (0.76. 1.41) | 0.817 |
|  | Model 3 | 1.08 (0.87. 1.33) | 0.49 |  | 0.95 (0.73. 1.24) | 0.712 | 1.01 (0.76. 1.35) | 0.937 |  | 0.90 (0.63. 1.28) | 0.544 |
|  | Model 4 | 1.05 (0.85. 1.31) | 0.661 |  | 0.94 (0.72. 1.22) | 0.638 | 1.02 (0.76. 1.36) | 0.905 |  | 0.90 (0.63. 1.28) | 0.545 |
| Abbreviations: CI, confidence interval; HR, hazard ratio; MET, metabolic equivalent of task; PREDIMED, PREvención con DIeta MEDiterránea; BMI, body mass index; CHD, coronary heart disease; CVD, cardiovascular disease; T2D, type 2 diabetes.  Model 1: adjusted for age (years), sex, and propensity scores; stratified by intervention group and recruitment center.  Model 2: model 1 + BMI, smoking status (never, former, or current smoker), alcohol intake and squared alcohol intake (grams per day), education level (primary, secondary, academic), physical activity (MET-min/day), family history of CHD (yes/no), hypertension (yes/no), hypercholesterolemia (yes/no), use of medication for lowering cholesterol(yes/no) and use of antihypertensive medication (yes/no),  Model 3: model 2 +, intake of total meat, fish, vegetables, fruits, cereals, dairy, nuts, and olive oil (g/day).  Model 4: model 3 + total legumes (g/day) from which the metabolite set was derived.  All dietary variables were adjusted for total energy intake using the residual method.  ^a^Analysis of CVD risk was performed among the participants of the PREDIMED CVD case–cohort data set or in the T2D case–cohort data set. Cox proportional hazard models, with Barlow weights, were used to estimate HRs and their 95% CIs for CVD or T2D. Person-time of follow-up was estimated as the interval between the baseline date and date of CVD or T2D event, death, or date of the last participant contact, whichever came first, HRs refer to 1-SD increase in correlated multimetabolite score.  ^b^Legume consumption, metabolic signatures, and covariates were assessed at year 1, and outcome was the incident CVD events occurred after the year 1 visit through the end of follow-up. The analytic models were the same as in the baseline analysis. | | | | | | | | | | | |

| **Additional table 7.**  Legume metabolite profile and risk of type 2 diabetes and cardiovascular disease by intervention groups | | | | | | | | | | | |
| --- | --- | --- | --- | --- | --- | --- | --- | --- | --- | --- | --- |
|  |  | **Control^c^** | | | | | **Intervention^d^**  **(Both MedDiet groups)** | | | | |
|  |  | **PREDIMED**  **Baseline^a^** | |  | **PREDIMED**  **1 year^b^** | | **PREDIMED**  **Baseline^a^** | | **PREDIMED**  **1 year^b^** | | |
|  |  | **HR**  **(95% CI)** | ***p*** |  | **HR**  **(95% CI)** | ***p*** | **HR**  **(95% CI)** | ***p*** |  | **HR**  **(95% CI)** | ***p*** |
| ***Type 2 diabetes*** | |  |  |  |  |  |  |  |  |  |  |
|  | No. of cases/total participants | **88/306** |  |  | **60/226** |  | **157/617** |  |  | **101/478** |  |
|  | Score of metabolites predicting legumes | |  |  |  |  |  |  |  |  |  |
|  | Model 1 | 0.92 (0.60, 1.43) | 0.723 |  | 1.05 (0.61, 1.83) | 0.851 | 0.83 (0.66, 1.04) | 0.099 |  | 0.98 (0.71, 1.34) | 0,884 |
|  | Model 2 | 0.93 (0.57, 1.51) | 0.768 |  | 1.03 (0.53, 1.98) | 0.935 | 0.70 (0.54, 0.91) | 0.008 |  | 0.95 (0.68, 1.35) | 0,79 |
|  | Model 3 | 0.98 (0.58, 1.64) | 0.928 |  | 0.84 (0.41, 1.71) | 0.62 | 0.68 (0.52, 0.90) | **0.006** |  | 0.94 (0.64, 1.38) | 0,747 |
|  | Model 4 | 1.00 (0.58, 1.73) | 0.995 |  | 0.84 (0.42, 1.68) | 0.62 | 0.69 (0.53, 0.91) | **0.007** |  | 0.93 (0.64, 1.36) | 0,714 |
| **Cardiovascular**  **disease** | |  |  |  |  |  |  |  |  |  |  |
|  | No. of cases/total participants | **77/307** |  |  | **96/421** |  | **145/686** |  |  | **108/642** |  |
|  | Score of metabolites predicting legumes | |  |  |  |  |  |  |  |  |  |
|  | Model 1 | 1.00 (0.89, 1.13) | 0.942 |  | 1.00 (0.89. 1.13) | 0.942 | 1.00 (0.89, 1.13) | 0.942 |  | 1.00 (0.89, 1.13) | 0.942 |
|  | Model 2 | 1.30 (0.96, 1.76) | 0.089 |  | 0.97 (0.75. 1.26) | 0.807 | 1.00 (0.83, 1.21) | 0.994 |  | 1.02 (0.83, 1.27) | 0.833 |
|  | Model 3 | 1.19 (0.87, 1.63) | 0.284 |  | 0.95 (0.73. 1.24) | 0.712 | 0.97 (0.79, 1.18) | 0.736 |  | 1.02 (0.82, 1.28) | 0.845 |
|  | Model 4 | 1.18 (0.84, 1.64) | 0.34 |  | 0.94 (0.72. 1.22) | 0.638 | 0.95 (0.78, 1.16) | 0.621 |  | 1.03 (0.83, 1.30) | 0.77 |
|  | Abbreviations: EVOO, extra virgin olive oil; CI, confidence interval; HR, hazard ratio; MET, metabolic equivalent of task; PREDIMED, PREvención con DIeta MEDiterránea; BMI, body mass index; CHD, coronary heart disease; CVD, cardiovascular disease; MedDiet: Mediterranean diet; T2D, type 2 diabetes.  Model 1: adjusted for age (years), sex, and propensity scores; stratified by intervention group and recruitment center.  Model 2: model 1 + BMI, smoking status (never, former, or current smoker), alcohol intake and squared alcohol intake (grams per day), education level (primary, secondary, academic), physical activity (MET-min/day), family history of CHD (yes/no), hypertension (yes/no), hypercholesterolemia (yes/no), use of medication for cholesterol (yes/no) and use of antihypertensive medication (yes/no),  Model 3: model 2 +, intake of total meat, fish, vegetables, fruits, cereals, dairy, nuts, and olive oil (g/day).  Model 4: model 3 + total legumes (g/day) from which the metabolite set was derived.  All dietary variables were adjusted for total energy intake using the residual method.  ^a^Analysis of CVD risk was performed among the participants of the PREDIMED CVD case–cohort data set or in the T2D case–cohort data set. Cox proportional hazard models, with Barlow weights, were used to estimate HRs and their 95% CIs for CVD or T2D. Person-time of follow-up was estimated as the interval between the baseline date and date of CVD or T2D event, death, or date of the last participant contact, whichever came first, HRs refer to 1-SD increase in correlated multimetabolite score.  ^b^Legume consumption, metabolic signatures, and covariates were assessed at year 1, and outcome was the incident CVD events occurred after the year 1 visit through the end of follow-up. The analytic models were the same as in the baseline analysis.  ^c^ Legume consumption (g/day) for low-fat diet control group: at baseline, T2D: 21 and CVD: 18; at one 1 year, T2D: 24 and CVD, 22.  ^d^ Legume consumption (g/day) for the MedDiet with EVOO and the MedDiet with nuts group: at baseline, T2D: 20 and CVD: 21; at one 1 year, T2D: 12 and CVD, 13. | | | | | | | | | | |
